# Imitation of St. John Long's Liniment

**Published:** 1831-01-01

**Authors:** 


### St. John Long's Liniment.

Two correspondents of the Medical Gazette, "who have been endeavouring conjointly to find out the panacea" of the renowned lung doctor of Harley Street, state that the following liniment produces precisely the same effects as that of Mr. Long.

℞. Acid. nitr. muriat. ℥ij.

Ol. terebinth . . ℥j.

— Camphoræ . . ℥v.

Misce ft. linimentum; — or, which is better :—

℞. Acid. nitr. muriat. ℥ij.

Ol. terebinth. . . ℥j.

Axungiæ . . . ℥v.

Melt the lard, and then add the other ingredients, stirring the whole till quite cold.

"Either of these liniments rubbed upon the skin with a sponge, will, in three or four minutes, cause it to be highly reddened, and the capillary vessels to be injected with blood. If the rubbing be continued, small pustules, here and there, containing a transparent limpid fluid, will make their appearance ; and if it be still persisted in, the part becomes excoriated, and an exudation of a thin clear lymph is the result. During its operaration, the patients first feel warmth in the part where it is applied, then smarting, and at length actual pain. With regard to the humour Mr. L pretends to extract, this appears to be nothing but the residue of the liniment left upon the skin ; and as to its affecting the part only where the disease is situated, it certainly is true that it does not seem to act upon the whole of the surface of the skin on the chest alike, but this arises from some parts being more tender than others ; hence the skin on the superior parts of the chest requires less rubbing with the liniment than that covering the ribs."

Without attaching the least credit to the supposition that this is the liniment employed by Long, we may ask, is the application of such a preparation as that above-described, upon the skin, of any service in phthisis, or not? We have tried it in several cases, and in all, the patients have expressed relief after its employment. They have had less cough, they have breathed more freely, and the expectoration has been diminished in quantity. We have only employed this remedy six weeks, and hardly that; so that what permanent good may result from it we know not. We cannot, however, but acknowledge that we think it might be a useful addition to the means we already possess, to produce *immediate* counter-irritation. We are fully aware that a blister is attended by a like result, but, at the same time, its action is slow, and its after-effect is such that it is some time before it can be repeated, and it cannot be applied over any extent of surface. The antimonial ointment, likewise, is another counter-irritant; but this also is uncertain in its action, and slow in its operation, and it cannot well be used again until the pustules arising from it have healed. The nitro-muriatic acid liniment, however, produces the effect almost instantaneously, and can be employed on any extent of surface without those inconveniences; for although it gives rise, at the time to a profuse determination of blood to the skin, yet this gradually subsides, and it can be re-applied (if it has not been improperly used) as often as we may consider necessary."

P.S. In the first of the forms published in our contemporary, a mechanical separation of the ingredients takes place on standing. The second formula is better adapted for use; but, unless a greater proportion than one half of the muriatic acid be used, the effect on the skin is inconsiderable. We have used the liniment on several occasions. It is a counter-irritant of easy application, and not very painful in its effects. We do not believe that it is at all similar to that which St. John Long employs. It may be found a useful remedy.

